# Association of Urinary Sodium, Potassium, and the Sodium-to-Potassium Ratio with Impaired Kidney Function Assessed with 24-H Urine Analysis

**DOI:** 10.3390/nu16193400

**Published:** 2024-10-07

**Authors:** Urte Zakauskiene, Nomeda Bratcikoviene, Ernesta Macioniene, Lina Zabuliene, Diana Sukackiene, Ausra Linkeviciute-Dumce, Dovile Karosiene, Valdas Banys, Vilma Migline, Algirdas Utkus, Marius Miglinas

**Affiliations:** 1 Institute of Clinical Medicine, Faculty of Medicine, Vilnius University, 03101 Vilnius, Lithuania; 2 Vilnius University Hospital Santaros Klinikos, 08661 Vilnius, Lithuania; 3 Institute of Biomedical Sciences, Faculty of Medicine, Vilnius University, 03101 Vilnius, Lithuania; 4 Faculty of Fundamental Sciences, Vilnius Gediminas Technical University, 10223 Vilnius, Lithuania; 5 Community Well-Being Center, Mykolas Romeris University, LT-08303 Vilnius, Lithuania

**Keywords:** sodium, potassium, sodium-to-potassium ratio, albumin excretion rate, AER, albuminuria, 24-h urine collection

## Abstract

**Background:** Albuminuria and albumin excretion rate (AER) are important risk factors for chronic kidney disease (CKD) development. Despite the extensive evidence of the influence of sodium and potassium on cardiovascular health, the existing evidence regarding their impact on albuminuria and kidney disease is limited and inconsistent. Our study aimed to assess the correlation between urinary sodium and potassium excretion, and the sodium-to-potassium ratio (Na/K ratio) with impaired kidney function, particularly the AER and albuminuria. **Materials and Methods:** Data were collected from the Lithuanian NATRIJOD study. A total of 826 single 24-h urine samples from individuals aged 18 to 69 were collected and analyzed for their sodium and potassium levels, Na/K ratio, and AER. Albuminuria was defined as an AER exceeding 30 mg/24 h. **Results:** The participant mean age was 47.2 ± 12.1 years; 48.5% of the participants were male. The prevalence of albuminuria was 3%. Correlation analysis revealed a positive correlation between AER and urinary sodium excretion (r_s_ = 0.21; *p* < 0.001) and urinary potassium excretion (r_s_ = 0.28; *p* < 0.001). In univariate linear regression analysis, sodium and potassium excretion and the Na/K ratio were significant AER predictors with *β* coefficients of 0.028 (95% CI: 0.015; 0.041; *p* < 0.001), 0.040 (95% CI: 0.003; 0.077; *p* = 0.035), and 1.234 (95% CI: 0.210; 2.259; *p* = 0.018), respectively. In the multivariable model, only urinary sodium excretion remained significant, with a *β* coefficient of 0.028 (95% CI: 0.016; 0.041). Potential albuminuria predictive factors identified via univariate logistic regression included urinary sodium excretion (OR 1.00; 95% CI: 1:00; 1.01) and the Na/K ratio (OR 1.53; 95% CI: 1.11; 2.05). However, these factors became statistically insignificant in the multivariate model. **Conclusions:** Urinary sodium and potassium excretion and the Na/K ratio are significantly associated with kidney damage, considering the assessed 24-h albumin excretion rate and presence of albuminuria content.

## 1. Introduction

Chronic kidney disease (CKD) is one of the non-communicable diseases affecting over 10% of the global population [1]. In the past 30 years, CKD prevalence has increased by 29.3%, and The Global Burden of Disease study in 2017 identified CKD as the 12th leading cause of death worldwide [2]. CKD prevalence varies across different socio-demographic regions, ages, and sex and is anticipated to become the fifth leading cause of lost years of life by 2040 [1,3,4,5]. CKD remains a major public health issue; thus, efficient prevention strategies are urgently needed [6].

Albuminuria is known as a predictive risk factor for the development of diabetes, cardiovascular disease, and damage to target organs. It is also a significant impaired kidney function marker [7]. While urinary albumin excretion can manifest in a “healthy” population, it usually indicates renal or combined renal and cardiovascular diseases. Overlapping pathologies and shared risk factors with cardiovascular disease contribute to important insights into albuminuria [7,8,9].

High sodium and low potassium consumption significantly affect cardiovascular morbidity and mortality [10,11]. Additionally, excessive sodium intake habits combined with low potassium intake and various pathophysiological, genetic, or environmental factors can contribute to target organ damage, including kidney disease development or progression, regardless of blood pressure levels [8,12,13,14,15,16,17]. Limiting salt consumption is a basic and cost-effective strategy for lowering mean arterial pressure, decreasing albuminuria, and reducing the risk of kidney function impairment [8,17,18,19]. Moreover, increased potassium consumption yields a reno-protective effect: each additional 1.0 g/day intake of urinary potassium is significantly associated with a 29% reduction in albuminuria risks [15]. The World Health Organization (WHO) recommends a daily consumption of less than 2000 mg of sodium (or less than 5 g of salt) and over 3510 mg of potassium for adults [20,21]. However, recent research on 24-h urinary electrolytes from US and European populations reveals that these recommended levels are not being met, with mean sodium and potassium consumption often exceeding twice the recommended amount and remaining insufficient, respectively [22,23,24,25,26].

The INTERSALT study emphasized the significance of lowering the sodium-to-potassium ratio (Na/K ratio) rather than just reducing sodium or increasing potassium consumption [27]. The WHO guidelines for dietary sodium and potassium intake [20,21] recommend the Na/K ratio of ≤0.6 mg/mg (≤1.0 mmol/mol by urinary excretion) [24]. An elevated Na/K ratio is associated with a significantly increased risk of kidney function decline, cardiovascular morbidity, and mortality, whereas lower ratios indicate a decreased risk of developing CKD [12,28,29,30]. Each unit increase in the Na/K ratio is associated with a 2.63% progression in urine albumin excretion [8].

There are still ongoing discussions regarding the best method in the field; however, 24-h urine collection remains the gold standard for assessing sodium, potassium, and albumin excretion [31,32,33].

This research used the baseline data from the Lithuanian NATRIJOD study [23] to evaluate the association between sodium and potassium excretion, the Na/K ratio, and impaired kidney function. This study particularly examined the albumin excretion rate (AER) and the presence of albuminuria using a 24-h urine collection technique.

## 2. Materials and Methods

### 2.1. Study Design

The Vilnius Region Bioethics Committee provided ethical approval (158200˗18/12˗1083˗580) for the NATRIJOD study, which is part of the broader initiative “Assessment of sodium and iodine status in the Lithuanian population and development of public health policy guidelines”. The project was supported by Vilnius University, Vilnius University Hospital Santaros Klinikos, the State Public Health Promotion Fund, and the WHO Regional Office in Europe. The Department of Statistics in Lithuania provided the demographic data. This study was carried out in accordance with the Declaration of Helsinki and Good Clinical Practice guidelines.

To assemble a nationally representative sample, healthy volunteers aged 18–69 were selected using random proportional sampling based on sex, age, and residency across the country’s three regions (North, Southeast, and West). The exclusion criteria are outlined in Table 1 [34,35,36]. Participants with self-reported current or past kidney disease and albuminuria levels exceeding 300 mg/24 h [8] were excluded from further analysis to ensure the reliability of the results. Participant recruitment and data collection continued from September 2019 to November 2020. Written informed consent was obtained from all willing participants.

### 2.2. Data Collection

Participants were invited during family physician appointments or through phone calls. Trained researchers and field workers collected the data. Each participant, after signing an informed consent form, was assigned a bar code (without personally identifiable data to ensure anonymity) and received a self-reported questionnaire, detailed verbal and written instructions for collecting a single 24-h urine sample, and plastic containers for 24-h urine collection.

All participants completed the questionnaire (which included 39 questions covering demographic, social, and health status, salt consumption and dietary behaviors, physical activity, and smoking habits). They underwent height, weight, blood pressure, and heart rate measurements using validated equipment and standardized protocols, as referenced in prior studies [23,25,26,34,36]. Smoking status was defined as smoking at least one cigarette per day. Physical activity was defined as engaging in physical activity that increased the pulse and respiration rate for 30 min per day, at least three times a week. Hypertension was defined as a blood pressure of 140/90 mmHg or higher or being on antihypertensive medication [36]. The presence of albuminuria was confirmed if the albumin excretion rate was found to be > 30 mg/24 h [37]. Body mass index (BMI) was calculated by dividing weight in kilograms by height in meters squared. Participants were divided in categories according to BMI: underweight (BMI < 18.5 kg/m^2^), normal weight (BMI 18.5–24.9 kg/m^2^), overweight (BMI 25.0–29.9 kg/m^2^), and obese (BMI ≥ 30 kg/m^2^) [38].

Laboratory evaluations were conducted following the submission of completed questionnaires and containers holding single 24-h urine samples to the healthcare facility.

Quality assurance standards were applied to avoid including invalid urine collections (Table 1). Biochemical urine parameters (sodium, potassium, chloride, albumin, creatinine, and urea) were analyzed using the Abbott Architect ci8200 analyzer, with dedicated reagents supplied by the same manufacturer (Abbott, Chicago, IL, USA) (Table 2).

### 2.3. Statistical Analysis

The data were analyzed using various statistical methods: descriptive and graphical data analysis to estimate the central tendencies of and variability in the total sample and specific groups; χ^2^ and Fisher Exact tests to identify proportional differences; the *t*-test and its non-parametric analog, the Wilcoxon signed-rank test, to compare means; analysis of variance (ANOVA) and the non-parametric Kruskal–Wallis test to assess group differences; Spearman correlation to evaluate associations and dependency strength between two continuous variables; and univariate linear regression models to evaluate the linear relationship. Only variables that remained statistically significant in univariate linear regression analysis were selected and input to the multivariable linear regression model. The methods used were determined by analyzing the objectives and fulfillment of parametric test assumptions. Statistical analysis and data processing were conducted using the R Project for Statistical Computing (version 4.2.2 (31 October 2022)) and RStudio (version 2023.12.1+402 “Ocean Storm” Release).

The urinary excretions of sodium and potassium (NaU and KU in mmol/24 h, respectively) were converted to daily intake estimates. These were calculated using the following equivalences: 1 mmol = 22.99 mg of sodium; 1 mmol = 39.1 mg of potassium. The sodium amount was multiplied by 2.542 to estimate salt (NaCl) intake from dietary sodium (Na). The sodium figures were then adjusted by a factor of 1.05, based on the assumption that approximately 95% of ingested sodium is excreted [39]. For potassium dietary intake, 85% of the ingested potassium was presumed to be excreted in urine [40].

## 3. Results

### 3.1. Baseline Characteristics

**Figure 1:**
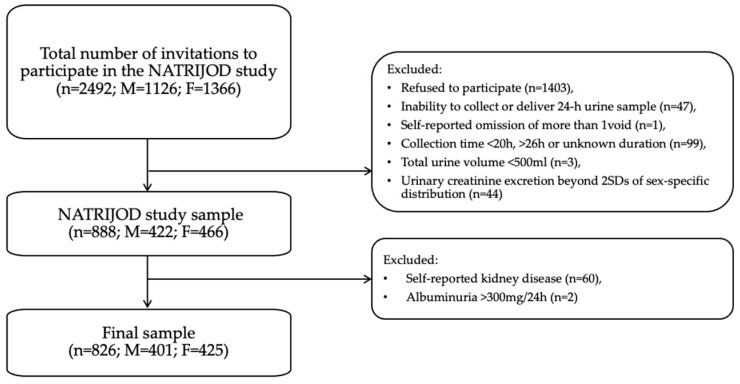
Flowchart of the study. *n*—number of participants, M—male, F—female.

Following the adjustment of additional exclusion criteria associated with albuminuria and kidney diseases, the final analysis included 826 individuals from the NATRIJOD study (Figure 1).

The mean participant age was 47.2 ± 12.1 years, and 48.5% of participants were male. Additionally, 3% of participants had albuminuria, and the median urinary albumin excretion rate was 5.8 mg/24 h (95% CI: 3.05, 8.97). Individuals with albuminuria were more likely to be male and had a significantly higher weight (89.5 kg vs. 79.5 kg, *p* = 0.002) and BMI (28.7 kg/m^2^ vs. 26.4 kg/m^2^, *p* = 0.008) than the albuminuria-negative group. Additionally, higher prevalences of overweight (33.9% vs. 56.0%, *p* = 0.01) and obesity (21.8% vs. 32.0%, *p* = 0.001) were determined in the albuminuria-positive group.

Additionally, the albuminuria-positive group had significantly higher systolic blood pressure (132.3 mmHg vs. 123.8 mmHg, *p* = 0.002) and increased diastolic blood pressure (81.4 mmHg vs. 77.8 mmHg, *p* = 0.079). A significantly larger percentage of participants with albuminuria were current smokers (44.0% vs. 16.1%, *p* < 0.001) and had diabetes (16.0% vs. 2.6%, *p* = 0.001). The baseline characteristics of the participants are outlined in Table 3.

Albuminuria-positive participants had significantly higher mean urinary sodium excretion (208.2 mmol/24 h vs. 161.9 mmol/24 h, *p* = 0.024), Na/K ratios (2.9 vs. 2.3, *p* = 0.016), and AERs (77.3 mg/24 h vs. 6.7 mg/24 h, *p* < 0.001) than the albuminuria-negative group, as shown in Table 4.

### 3.2. AER Levels across the Quartiles of Urinary Sodium and Potassium Excretion and the Na/K Ratio

The 24-h urinary sodium excretion quartiles (mmol/24 h) were 106.5, 146.3, and 199.4. The urinary potassium excretion quartiles (mmol/24 h) were 52.9, 69.9, and 88.3. The Na/K ratio quartiles were 1.6, 2.1, and 2.9.

**Figure 2:**
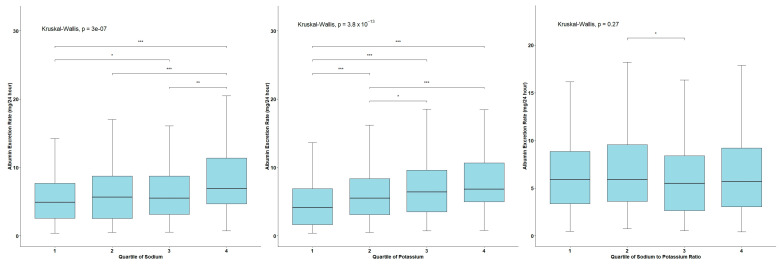
The 24-h urinary albumin excretion rate box plots for the sodium, potassium, and Na/K ratio quartiles. The value 1 corresponds to interval (min, Q_1_], 2—(Q_1_, Q_2_], 3—(Q_2_, Q_3_], and 4—(Q_3_, max). The lines at the top of the plot highlight statistically significant differences between quartile groups as determined using the Kruskal–Wallis test. Significance levels are indicated as follows: * *p*-value < 0.05, ** *p*-value < 0.001, *** *p*-value < 0.0001.

The 24-h urinary AER box plots across sodium, potassium, and Na/K ratio quartiles are presented in Figure 2. We noticed the median AER tendency to increase with urinary sodium and potassium excretion quartiles. Participants in the highest sodium and potassium excretion quartiles had significantly higher AER than the lower quartiles. However, no significant trend was observed in AER across the Na/K ratio quartiles.

Significant differences in AER distributions were found between the following urinary sodium excretion values: (min, Q_1_] vs. (Q_2_, Q_3_] (*p* = 0.03); (min, Q_1_] vs. (Q_3_, max) and (Q_1_, Q_2_] vs. (Q_3_, max) (*p* < 0.0001); and (Q2, Q3] vs. (Q_3_, max) (*p* < 0.0001). For urinary potassium excretion, significant differences were observed between values in the following intervals: (Q_1_, Q_2_] vs. (Q_2_, Q_3_] (*p* = 0.04); (min, Q_1_] vs. (Q_1_, Q_2_]; (min, Q_1_] vs. (Q_2_, Q_3_]; (min, Q_1_] vs. (Q_3_, max); and (Q_1_, Q_2_] vs. (Q_3_, max) (*p* < 0.0001). For the urinary Na/K ratio, significant differences were observed between values in the intervals of (Q_1_, Q_2_] vs. (Q_2_, Q_3_] (*p* < 0.05).

### 3.3. Correlation Analysis between Urinary Sodium and Potassium Excretion, the Na/K Ratio, and AER

**Figure 3:**
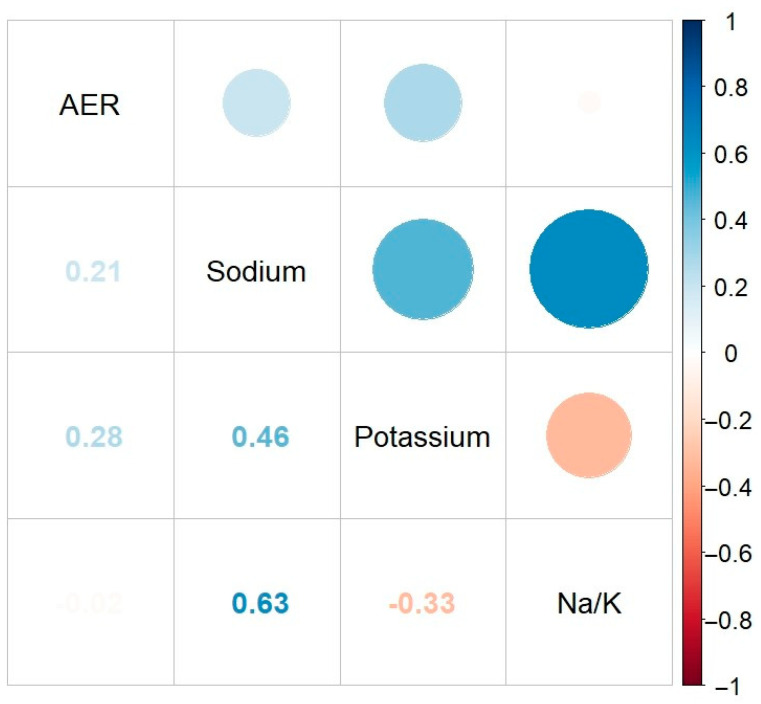
Correlogram showing the pairwise correlations between urinary sodium and potassium excretion, the Na/K ratio, and AER. Positive and negative correlations are represented in blue and red, respectively. Color intensity and circle size are proportional to the correlation coefficients. Na/K ratio—sodium-to-potassium ratio, AER—albumin excretion rate.

Spearman correlation coefficient and univariate linear regression models were applied to analyze the correlation between urinary sodium, potassium, the Na/K ratio, and AER. The multivariable linear regression model included only those variables that remained statistically significant in the univariate analysis. Spearman correlation analysis identified positive low-to-moderate correlations between AER and urinary sodium excretion (r_s_ = 0.21, *p* < 0.001) and urinary potassium excretion (r_s_ = 0.28, *p* < 0.001). However, no significant correlation was found between the Na/K ratio and AER (Figure 3).

### 3.4. Estimates of Linear and Logistic Regression Models

Table 5 represents the linear regression model results predicting AER. An increase in each unit in urinary sodium excretion (mmol/24 h) is associated with a 0.03 mg/24 h increase in AER (95% CI: 0.015, 0.041). Both urinary potassium excretion and the Na/K ratio are also statistically significant predictors of AER, with increases of 0.04 mg/24 h (95% CI: 0.003, 0.077; *p* = 0.035) and 1.23 mg/24 hour (95% CI: −0.21, 2.259; *p* = 0.018), respectively. Other factors, including sex, weight, BMI, systolic and diastolic blood pressure, hypertension, and diabetes, also predicted a significant impact on AER. Only urinary sodium excretion and diabetes remained significant albuminuria predictors in the multivariable model.

**Figure 4:**
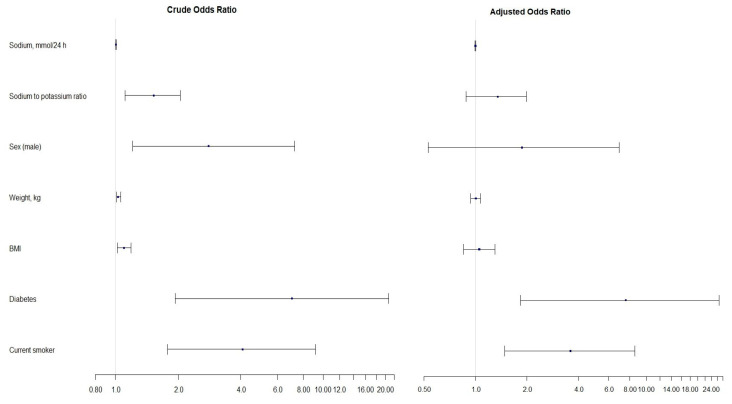
Forest plot showing the crude and adjusted odds ratios of predictors in the ordinal logistic regression models.

Possible albuminuria predictors were identified by applying univariate logistic regression models. Factors that remained statistically significant in univariate binary logistic regression were included in the multivariable binary logistic regression model. The odds ratio (OR) and 95% confidence intervals (95% CI) are presented in Table 6. The forest plot with the odds ratios from both univariate and multivariable ordinal logistic regression models is shown in Figure 4. The horizontal error bars indicate 95% confidence intervals.

Urinary sodium excretion, the Na/K ratio, sex (male), weight, BMI, diabetes, and smoking status were identified as significant AER predictors in the univariate analysis (Table 6). History of diabetes was associated with more than seven-time higher odds of albuminuria (OR 7.07; 95% CI: 1.94, 20.66). Smoking increased the odds of albuminuria by more than four times (OR 4.09; 95% CI: 1.78, 9.20). These two factors remain significant in the multivariable model. After adjusting for covariates, the OR increased to 7.55 (95% CI: 1.83, 26.63, *p* = 0.003) and 3.59 (95% CI: 1.48, 8.56, *p* = 0.004) for diabetes and smoking, respectively. However, the wide confidence intervals suggest that the estimates may be unreliable due to potential under-sampling.

## 4. Discussion

Our population-based cross-sectional study evaluated the associations between urinary electrolyte excretion and kidney function in a healthy Lithuanian population, with a focus on AER and the presence of albuminuria. Participants with albuminuria tend to be male and smokers and have conditions such as diabetes, higher weight, BMI, and systolic blood pressure. The mean AER was 77.3 mg/24 h. A positive association was detected between urinary sodium excretion and AER and between potassium excretion and AER. Nonetheless, after adjusting models for regression analysis, no significant association was found between the Na/K ratio and the present albuminuria.

The prevalence of albuminuria varies in the literature. The PREVEND study reported that 7.2% of participants had microalbuminuria [41]. The REGARDS study found albuminuria prevalence rates of 11.5%, 11.6%, and 16% in normal-weight, overweight, and obese individuals, respectively [42]. The Korea National Health and Nutrition Study indicated that 5.2% of the general population and only 2.1% of non-diabetic, non-hypertensive individuals had albuminuria [43]. In our study, albuminuria incidence was determined to be in 3% of the sample Lithuanian population of 826 participants.

Albuminuria, as a sign of kidney function impairment, is associated with high salt consumption and increased urinary sodium excretion [8,42,44,45]. A study from Switzerland by Deriaz et al. stated that higher sodium excretion is associated with a more rapid renal function decline [12]. Our study confirmed a significant positive correlation between AER and urinary sodium excretion (r_s_ = 0.21, *p* < 0.001). Consistent with our results, the China Smash survey identified a significant, independent association between urinary sodium excretion and albuminuria (r = 0.107, *p* < 0.001) regardless of cardiovascular disease risk factors, including systolic blood pressure [44]. Similarly, Sun et al. reported that higher salt consumption (fifth quintile) significantly increases the likelihood of albuminuria (OR 2.154; 95% CI: 1.431, 3.242)), even when adjusting for confounders such as BMI, systolic blood pressure, and others [8]. Chen et al.’s meta-analysis indicated that reducing salt intake restriction lowers blood pressure and decreases the urinary albumin excretion rate by 12.62 mg/min (95% CI: 19.64, 5.60) in diabetic kidney disease [46]. Xu et al. reported that each additional gram in sodium intake increases urinary albumin by 1.16 mg per day in China [47]. The PREVEND study from the Netherlands found that a 1 g increase in sodium intake over a prolonged period increases the urinary albumin excretion by 4.6 mg/d [41]. While significant, our findings were less remarkable than these studies. A primary NATRIJOD study found that average salt consumption in Lithuania is 10 g/d, which is twice the value recommended by the WHO [23]. In the current study, the linear univariate analysis revealed that each gram increase in sodium intake was associated with a 0.46 mg/24 hour increase in AER (95% CI: 0.252, 0.662, *p* < 0.001). This association remained significant in the multivariable model (*β* = 0.46; 95% CI: 0.259, 0.662, *p* < 0.001), unlike other factors, such as sex, weight, BMI, systolic and diastolic blood pressure, and hypertension. Urinary sodium excretion was identified as a significant prognostic factor for the presence of albuminuria in the univariate logistic regression model. Differences between studies may stem from various factors, such as cohort sample size, baseline characteristics, average salt consumption and mean urinary sodium excretion, methods, and study designs.

On the other hand, potassium protects the kidneys. Low urinary potassium excretion is associated with higher CKD risk [16]. Increasing potassium intake may lower the risk of rapid glomerular filtration rate (GFR) decline [29] and blood pressure in hypertensive patients [48]. The CARDIA study, which lasted 20 years, found that each 1 g increase in potassium consumption significantly reduces the risk of albuminuria [15]. Similarly, Yuan et al. reported that higher potassium consumption is associated with a reduced risk of albuminuria, specifying that each unit increment in potassium intake is associated with a 1% decrease in the risk of developing proteinuria [49]. However, findings across studies are inconsistent. Some research reported no significant associations between potassium intake and albuminuria [44,47]. It is important to note many of these studies were conducted in China, where average potassium consumption is exceptionally low, around 1.6 g/d [44], significantly below the WHO’s recommendation. The difference in potassium intake levels may have influenced the results of the studies mentioned above.

In our study, we observed a significant upward trend in albumin excretion rate across potassium excretion quartiles (*p* < 0.001) and a positive correlation between AER and urinary potassium excretion (r_s_ = 0.28, *p* < 0.001). Urinary potassium excretion proved to be a significant prognostic AER factor in the univariate linear regression model, with each unit increase in urinary potassium leading to a 0.04 mg/24 hour increase in AER (95% CI: 0.003, 0.077; *p* = 0.035).

The WHO recommends a daily potassium intake exceeding 3.5 g/24 h [21]. The NATRIJOD study found that the mean potassium intake in Lithuania is 3.3 g/24 h (with men consuming 3.6 g/24 h, and women 3.1 g/24 h) [23]. Unpublished data from the same study show that over 40% and 38% of participants consume fresh vegetables and fruits more than six times a week, respectively [23]. Also, the higher salt-rich food intake, increased salt use in cooking, frequent mineral or spring water consumption, and a higher vegetable and fruit intake were documented [23]. This might explain the observed increase in AER across potassium quartiles and the significant correlation between potassium excretion and albuminuria in the present study. Additionally, a significant association between urinary sodium excretion and albuminuria was found. Despite the tendency to add salt during cooking and a preference for salty foods, which may increase the likelihood of albuminuria, participants also consumed potassium-rich products and beverages. Nonetheless, fresh vegetable and fruit consumption in Lithuania is inadequate, while the potassium intake is nearly adequate. Compared to other countries, where potassium intake is often more than twice below the recommended amount [4,8,44], Lithuania’s intake is comparatively higher, and this finding could explain the relatively low prevalence (3%) of albuminuria.

According to the WHO guidelines [20,21], the recommended Na/K ratio is ≤0.6 g/g, and the recommended molar Na/K ratio is ≤1.0 mmol/mmol. Sun et al. found a positive correlation between the Na/K ratio and albuminuria [8]. Two recent studies discovered that a high urinary Na/K ratio is associated with a faster decline in kidney function among adults in the general population [12,29]. Similarly, Koo et al. reported an association between higher urinary Na/K ratio and CKD progression risk [30]. An epidemiological and genome study in Korea found that subjects in the lowest tertile, according to the urinary Na/K ratio, have a significantly lower risk of developing CKD than the highest tertile. In the fully adjusted model, the hazard ratio (HR) of the lowest to highest tertile was 0.78 (95% CI, 0.63–0.97), indicating a 22% lower risk [28]. In our study, the correlation between the Na/K ratio and AER was not significant. However, the Na/K ratio was a significant prognostic AER factor in the univariate regression model: for each unit increase in the Na/K ratio, AER increased by 1.23 mg/24 h (95% CI: 0.21, 2.259; *p* = 0.018). In the univariate logistic regression, a higher Na/K ratio was associated with a 1.53 times greater risk of albuminuria (95% CI: 1.11, 2.05; *p* = 0.006). However, the Na/K ratio was not statistically significant in the fully adjusted model, and other studies have not confirmed this association [44,47]. Our study’s conclusions might be affected by the unexpectedly higher, nearly normal urinary potassium excretion compared to other studies, particularly those conducted in China [8,12,44,47].

Our study has several major strengths. First, the reliability and generalizability of our results increased because we used a randomly selected nationally representative sample. The gold-standard 24-h urine collection technique was selected to assess sodium, potassium, and albumin excretion. Spot urine tests offer less burden to participants but do not meet the accuracy of the 24-h collection method. We also implemented a quality control protocol, including detailed verbal and visual instructions of field workers during the first visit. Additionally, we validated samples according to laboratory exclusion criteria (eligible collection time, volume, and additional test for creatinine levels) and excluded participants with self-reported kidney disease or albuminuria >300 mg/24 h.

Nevertheless, our study had limitations. We collected only a single 24-h urine sample. More comprehensive information on long-term outcomes, personal behaviors, and individual variability would have been available from multiple samples. The complexity of the 24-h urine collection requires significant participant commitment and effort. While only one subject reported missing more than one void, it is possible that other under-collections were unreported, along with inaccurate or incomplete self-reported information on health status and medication usage. After setting thresholds for creatinine, urine volume, and collection time, we excluded 146 participants. We also did not collect blood samples, which would have allowed creatinine testing and identification of participants with CKD. Finally, the cross-sectional design of our study limits our capacity to determine a causal relationship between salt intake and albuminuria.

## 5. Conclusions

We found a correlation between urinary sodium excretion and impaired kidney function, characterized by 24-h albumin excretion rates and the presence of albuminuria. Our study also detected associations between potassium excretion levels, the Na/K ratio, and both AER and albuminuria. Although further investigation is necessary to confirm these results, our findings support current recommendations for the general population on sodium and salt intake reduction.

## Figures and Tables

**Table 1:** Study exclusion criteria.

Primary Exclusion Criteria	Secondary Exclusion Criteria	Laboratory Exclusion Criteria
Age (<18 or >69 years); Known history of stroke or heart, kidney, or liver diseases; Prescribed diuretic therapy within the last two weeks before urine sample collection; Terminally or mentally ill individuals; Pregnant women; Inability to collect 24-h urine sample; Unwillingness or inability to provide informed consent for study participation.	Inability to collect or deliver the collected urine sample; Self-reported omission of more than one void; Self-reported kidney disease.	Total urine volume < 500 mL; Collection time <20 h, >26 h, or of unknown duration; Urinary creatinine excretion beyond two standard deviations (SDs) of sex-specific distribution; Albuminuria > 300 mg/24 h.

**Table 2:** An overview of the methods used in the urine biochemistry panel and details about their traceability.

Analyte (Urine)	Method	Traceability to Reference Material
Creatinine	Photometric, enzymatic	NIST SRM 967
Sodium, potassium, chloride	Indirect potentiometry (indirect ion selective electrode (ISE))	NIST SRM 918 and NIST SRM 919
Urea	Photometric, enzymatic	NIST SRM 912
Albumin	Immunoturbidimetric	CRM 470

**Table 3:** Baseline characteristics of participants according to the presence of albuminuria.

Variables	All Participants	Albuminuria (−) Group	Albuminuria (+) Group	*p*-Value
*n*	826	801	25	
Sex: male, *n* (%)	401 (48.5)	383 (47.8)	18 (72.0)	<0.001 *
Age, year	47.2 (12.1)	47.1 (12.1)	50.8 (12.3)	0.138
Height, cm	173.4 (9.5)	173.3 (9.5)	176.3 (6.5)	0.056
Weight, kg	79.8 (16.6)	79.5 (16.5)	89.5 (15.9)	0.002
BMI, kg/m^2^	26.5 (4.6)	26.4 (4.6)	28.7 (4.7)	0.008
BMI, *n* (%)				0.01 **
<18.5 kg/m^2^	13 (1.6)	13 (1.7)	0 (0.0)	
18.5–24.9 kg/m^2^	335 (41.7)	332 (42.6)	3 (12.0)	
25.0–29.9 kg/m^2^	278 (34.6)	264 (33.9)	14 (56.0)	
≥30 kg/m^2^	178 (22.1)	170 (21.8)	8 (32.0)	
Systolic BP, mmHg	124.0 (22.9)	123.8 (23.1)	132.3 (15.3)	0.002
Diastolic BP, mmHg	77.9 (9.3)	77.8 (9.3)	81.4 (9.2)	0.079
Pulse rate, bpm	72.7 (10.1)	72.6 (10.2)	74.2 (9.0)	0.283
Hypertension, *n* (%)	284 (34.8)	272 (34.3)	12 (50.0)	0.170 *
Antihypertensive medication, *n* (%)	70 (8.5)	68 (8.5)	2 (8.0)	1.000 *
Diabetes, *n* (%)	25 (3.0)	21 (2.6)	4 (16.0)	0.001 *
Current smokers, *n* (%)	140 (16.9)	129 (16.1)	11 (44.0)	<0.001 *
Physical activity: active, *n* (%)	543 (65.8)	522 (65.2)	21 (84.0)	0.082 *

Results are displayed in this table as mean (SD) for continuous variables or as a percentage for categorical variables. *n*—number of participants, BMI—body mass index; BP—blood pressure. p-value indicates the statistical difference between groups according to the presence of albuminuria. * The *p*-value indicates the significance of the difference between groups based on the presence of albuminuria. ** Fisher’s exact test p-value is used. Elsewhere, the p-value of the Kruskal–Wallis rank sum test is used.

**Table 4:** The 24-h urine analysis includes urinary sodium and potassium excretion and the sodium-to-potassium ratio, categorized by the presence of albuminuria.

Variables	All Participants	Albuminuria (−) Group	Albuminuria (+) Group	*p*-Value
*n*	826	801	25	
Sodium, mmol/24 h	163.3 (86.4) 146.3 (106.5–199.4)	161.9 (85.1) 145.8 (106.4–197.5)	208.2 (114.3) 196.6 (124.5–299.0)	0.024
Potassium, mmol/24 h	73.8 (29.6) 69.9 (52.9–88.3)	73.8 (29.6) 69.9 (52.8–88.4)	72.1 (28.0) 72.3 (60.5–82.1)	0.871
Na/K ratio	2.3 (1.1) 2.1 (1.6–2.9)	2.3 (1.1) 2.1 (1.6–2.9)	2.9 (1.3) 2.9 (1.8–4.0)	0.016
Salt intake, g/24 h	10 (5.3) 9 (6.5–12.2)	9.9 (5.2) 8.9 (6.5–12.1)	12.8 (7.0) 12.1 (7.6–18.4)	0.024
Potassium intake, g/24 h	3.3 (1.3) 3.1 (2.4–4.0)	3.3 (1.3) 3.1 (2.4–4.0)	3.2 (1.3) 3.2 (2.7–3.7)	0.876
AER, mg/24 h	8.8 (16.1) 5.8 (3.1–9)	6.7 (5.1) 5.7 (3.0–8.6)	77.3 (55) 51.4 (38.5–102.8)	<0.001

Results are displayed in this table as mean (SD) and the median (25th and 75th percentiles) for continuous variables or as a percentage for categorical variables, *n*—number of participants, Na/K ratio—sodium-to-potassium ratio, AER—albumin excretion rate. *p*-value indicates the significance of the difference between groups based on the presence of albuminuria.

**Table 5:** Associations of 24-h urinary albumin excretion (mg/24 h) using linear regression models.

Factor	Univariate Model		Multivariable Model	
	Parameter β (95% CI)	*p*-Value	Parameter β (95% CI)	*p*-Value
Sodium, mmol/24 h	0.028 (0.015, 0.041)	<0.001	0.028 (0.016, 0.041)	<0.001
Potassium, mmol/24 h	0.040 (0.003, 0.077)	0.035	statistically insignificant	
Na/K ratio	1.234 (0.210, 2.259)	0.018	statistically insignificant	
Sex (male)	2.940 (0.747, 5.133)	0.009	statistically insignificant	
Weight, kg	0.130 (0.062, 0.197)	<0.001	statistically insignificant	
BMI, kg/m^2^	0.389 (0.147, 0.631)	0.002	statistically insignificant	
Systolic blood pressure, mmHg	0.054 (0.007, 0.102)	0.025	statistically insignificant	
Diastolic blood pressure, mmHg	0.153 (0.037, 0.270)	0.010	statistically insignificant	
Hypertension	4.135 (1.867, 6.404)	<0.001	statistically insignificant	
Diabetes	18.383 (12.082, 24.684)	<0.001	18.475 (12.246, 24.703)	<0.001

BMI—body mass index, Na/K ratio—sodium-to-potassium ratio, AER—albumin excretion rate.

**Table 6:** Factors associated with albuminuria using logistic regression models.

Factor	Crude		Adjusted	
	Odds Ratio (95% CI)	*p*-Value	Odds Ratio (95% CI)	*p*-Value
Sodium, mmol/24 h	1.00 (1.00, 1.01)	0.010	1.00 (1.00, 1.00)	statistically insignificant
Na/K ratio	1.53 (1.11, 2.05)	0.006	1.35 (0.88, 1.99)	statistically insignificant
Sex (male)	2.81 (1.21, 7.29)	0.022	1.87 (0.53, 6.93)	statistically insignificant
Weight, kg	1.03 (1.01, 1.06)	0.003	1.00 (0.94, 1.07)	statistically insignificant
BMI, kg/m^2^	1.10 (1.02, 1.19)	0.014	1.05 (0.85, 1.30)	statistically insignificant
Diabetes	7.07 (1.94, 20.66)	<0.001	7.55 (1.83, 26.63)	0.003
Smoking	4.09 (1.78, 9.20)	<0.001	3.59 (1.48, 8.56)	0.004

BMI—body mass index, Na/K ratio—sodium-to-potassium ratio.

## Data Availability

Data are available upon reasonable request in writing to the corresponding author.
